# The first large-scale All Taxa Biodiversity Inventory in Europe: description of the Mercantour National Park ATBI datasets

**DOI:** 10.3897/BDJ.10.e85901

**Published:** 2022-12-21

**Authors:** Jean Ichter, Olivier Gargominy, Marie-France Leccia, Solène Robert, Laurent Poncet

**Affiliations:** 1 Muséum national d'Histoire naturelle (correspondent), Paris, France Muséum national d'Histoire naturelle (correspondent) Paris France; 2 PatriNat (OFB/MNHN/CNRS), Paris, France PatriNat (OFB/MNHN/CNRS) Paris France; 3 Parc national du Mercantour, Nice, France Parc national du Mercantour Nice France

**Keywords:** Alps, biodiversity, chorology, conservation, ecology, France, hotspot, Italy, survey, taxonomy

## Abstract

**Background:**

An All Taxa Biodiversity Inventory (ATBI) is a comprehensive inventory of all species in a given territory. In 2007, the French Parc national du Mercantour and the Italian Parco Naturale Alpi Marittime started the first and most ambitious ATBI in Europe with more than 350 specialists and dozens of technicians and data managers involved.

**New information:**

The ATBI datasets from the Parc national du Mercantour in France are now publicly available. Between 2007 and 2020, 247,674 occurrences were recorded, checked and published in the INPN information system. All this information is available in open access in the GBIF web site. With 12,640 species registered, the ATBI is the most important inventory in France. This data paper provides an overview of main results and its contribution to the French National Inventory of Natural Heritage. It includes a list of 52 taxa new to science and 53 species new to France, discovered thanks to the ATBI.

## Introduction

The question of how many species belong to a given territory has always been an excellent driver for field biology studies and the starting point of many scientific findings (Kohler 2006, Bouchet et al. 2008). Despite centuries of species description, our knowledge on biodiversity is far from being complete, especially for the smallest organisms, such as invertebrates and non-vascular plants (Brown et al. 2018). Several authors acknowledge that these poorly-studied species are facing higher rates of extinction (McKinney 1999, Thomas et al. 2004, Sánchez-Bayo and Wyckhuys 2019) as part of a wider phenomenon: the Earth’s sixth major extinction event (Ceballos, Gerardo et al. 2015, Cowie et al. 2022). The idea that many species would disappear before being discovered has generated increasing support from society towards ambitious species inventories (Dubois 2003, Mauz 2011). Although less popular, the specialists capable of naming and classifying living organisms, taxonomists, are also in decline (Fontaine et al. 2012). The shortage of taxonomists and curators, known as the 'taxonomic impediment', was for the first time internationally recognised in 1992 in Rio de Janeiro at the Convention on Biological Diversity.

Originally developed by the American ecologist, Daniel Janzen, for a project in Costa Rica, the concept of All Taxa Biodiversity Inventory (ATBI) is an approach for completing a comprehensive survey of the plants and animals living in a natural (or semi-natural) area, including data on their environment (e.g. habitat, ecological niche), their abundance, behaviour and the genetic diversity (Deharveng et al. 2015b, White and Langdon 2006). The first large-scale ATBI in the Great Smoky Mountains National Park in the USA ([Bibr B5750868][Bibr B5760291]) showed unexpected results with 18,000 described species recorded and almost 1,000 species new to science (www.dlia.org). This experience inspired dozens of ATBI’s across the world (Ichter et al. 2018), including the first European ATBI between the French Parc national du Mercantour and the Italian Parco Naturale Alpi Marittime (De Biaggi et al. 2013). With more than 12,640 species described including 50 new to science, this project is the first and most successful large-scale All-Taxa Biodiversity Inventory in Europe.

These datasets are now available in open access in both national (https://openobs.mnhn.fr) and global (www.gbif.org) biodiversity facilities. The objective of this data paper is to provide an updated description of the datasets produced in the framework of the ATBI in the Mercantour National Park, an overview of main results and its contribution to the French National Inventory of Natural Heritage (https://inpn.mnhn.fr).

## General description

### Purpose

An All Taxa Biodiversity Inventory (ATBI) is a comprehensive inventory of all species occurring in a given territory. Its objective is to improve knowledge on taxonomy and chorology and to better understand ecological communities and their interactions within ecosystems (Janzen and Hallwachs 1994, Ichter et al. 2018). It encourages further data acquisition and collation of existing knowledge (e.g. historical data) to maximise the number of species inventoried and associated ecological information. Wherever feasible, it aims to contribute to better management of the territory through assessments and monitoring.

### Additional information

The ATBI Mercantour/Alpi Marittime project started with the creation of the European Distributed Institute of Taxonomy (EDIT) in 2006. EDIT was a network of excellence of 28 institutions whose main objective was to reduce the fragmentation in European taxonomy. With the support of the Muséum national d'Histoire naturelle (MNHN) in Paris, the Parc national du Mercantour and the Parco Naturale Alpi Marittime applied to host a pilot project called 'All taxa biodiversity inventory + monitoring' (ATBI+M) as the first of a series in Europe.

Thanks to their high potential for biodiversity, logistical opportunities and successful previous scientific partnerships (Gargominy and Ripken 2006, Hervé and Rollard 2009), EDIT chose 17 pilot sites within both parks. In 2008, a dataset of 31,680 occurrences was published in the GBIF (https://doi.org/10.15468/4z4hto) by EDIT's coordinators, the Museum für Naturkunde Berlin (MfN) and the Staatliches Museum für Naturkunde Stuttgart (SMNS).

In 2009, when the EDIT work package ended, the two natural parks in collaboration with the MNHN in Paris and the Museo Regionale di Scienze Naturali in Turin (MRSN) proposed to continue the project, not only in pilot sites, but to their entire territories and with increased outputs in terms of management and decision-making. A three-year project called *Inventaire Biologique Generalisé / Inventario Biologico Generalizzato* (Generalised Biological Inventory) was accepted as an Integrated Transboundary Action Plan in the framework of the ALCOTRA 2007-2013 programme. Both park administrations were responsible for the fieldwork coordination and the MNHN was tasked with data management through a web-based application (https://cardobs.mnhn.fr/).

Inventories continued after the end of the EU funded programmes (EDIT and ALCOTRA). Scientists and naturalists are still conducting fieldwork and investigations on lesser-known species. The park authorities provided authorisations and conventions in exchange for the transmission of the data. The Mercantour National Park also initiated several projects that are a direct continuity of the ATBI: Explor'Nature (bioblitz), Programme Abeilles Sauvages (Wild Wasps), Myriapods inventory and ABC (Communal Atlas of Biodiversity). A new transboundary Alpine ATBI, funded by PITEM Biodiv'ALP, started in 2019 and is further proof of the persistence of this dynamic (see https://www.interreg-alcotra.eu). The authors consider the ATBI Mercantour/Alpi Marittime as an on-going collective process and follow the Mauz and Granjou (2013) definition of an ATBI: a boundary institution in the sense of an assemblage of actors in motion with fuzzy boundaries.

## Project description

### Personnel

More than 350 individual specialists contributed to the ATBI. Additionally, dozens of park rangers actively helped with preparation, fieldwork or conducting inventories. Two project managers were recruited to coordinate the programme in each park. In the Mercantour, two seasonal field technicians were hired from 2009 to 2012 as support for fieldwork and to collate historical data. After the fieldwork, many students, volunteers and laboratory technicians were tasked with sorting a large amount of material especially for the continuous sampling techniques (e.g. entomological traps). Although resources were specifically dedicated to material sorting and data management, part of the information was not yet available due to insufficient resources: time, finance and available experts (Villemant et al. 2015). The preparation of this publication was an opportunity for a qualitative and quantitive update of the different ATBI datasets. Participants were contacted to update their data. In addition, a review was initiated to ensure that all data of recently-published papers (e.g. new species for science or France, taxonomic revision) were entered into the database.

### Study area description

**Figure 1.**
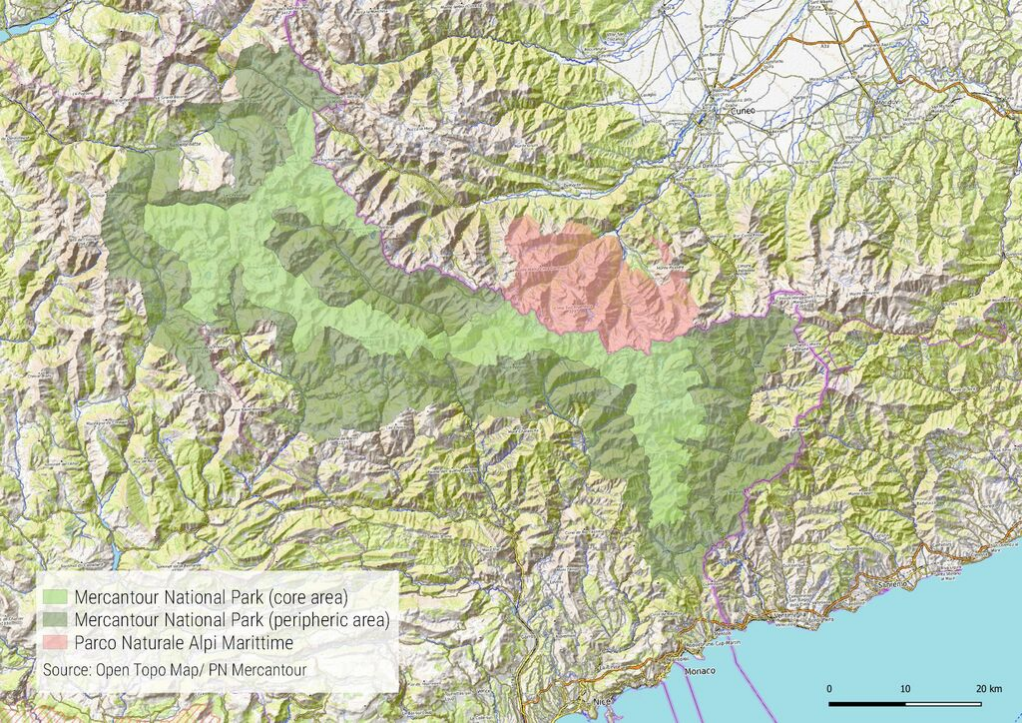
Map of the Parc national du Mercantour, France and the Parco Naturale Alpi Marittime, Italy (OpenTopoMap / Parc national du Mercantour, under CC-BY-SA).

The Mercantour National Park is part of the Mercantour- Argentea mountain range in the Southern Alps (Fig. 1). Ranging from 350 to 3,297 m a.s.l. (Mont Argentera), it is influenced by both Mediterranean and Alpine climates and is crossed by numerous rivers, the main ones being the Roya, the Bévéra, the Tinée, the Vésubie, the Var, the Cians, the Verdon and the Ubaye. It is also characterised by varied geology, a great diversity of habitats and climatic influences from the Mediterranean, Alpine and Ligurian Regions. The complex geology has created a great variety of bedrocks with very old crystalline rocks (gneiss, granite) and younger sedimentary rocks (juvenile karst, schist, sandstone). Moreover, the area was not affected by the last glacial period in the Alpine Region and served as a refugium for many species (Médail and Diadema 2009). This particular ecological and biogeographic situation is at the origin of a great diversity of ecosystems and life forms. The study area is part of a widely recognised hotspot of biodiversity in Europe (Medail and Quezel 1999, Dole-Olivier et al. 2015, Villemant et al. 2015).

### Design description

During the first two years of the project (2007-2009), the sampling strategy coordinated by EDIT was to concentrate the effort on an intensive survey of 17 pilot sites. In 2009, a transboundary Steering Committee was created and composed of around 10 people including Parks' staff, taxonomists, ecologists, a hydrobiologist and a biomathematician. The sampling effort was extended to the whole area of the two parks, to increase the diversity of habitat and the potential use in terms of management and monitoring. By increasing the area sampled, species richness and representativity also increased. However, as a result, not all areas could not be monitored as exhaustively as originally planned.

In terms of organisation, participants could work independently or within a coordinated thematic group. Independent specialists could decide the dates and the sites to visit. They were given permission by the park authorities to sample and could apply to have their fees reimbursed. In exchange, they agreed to liaise with park authorities to announce their visits, share information on their sampling methods, provide a report with the results of their research and deliver a specimen of each species collected. Due to the great number of taxonomists (350+), countries and institutions involved, the majority of field days were organised this way.

As the inventory progressed, the steering committee preferred the groups thematic system as used in the Smoky Mountains ATBI. They were built around different sampling strategies: 1) taxonomical targets (e.g. lichens, bryophytes, tracheophytes); 2) biological groups with similar sampling strategies (e.g. terrestrial invertebrates); or 3) types of ecosystems (e.g. superficial aquatic habitats, biospeology).

These two approaches (by groups and/or independent) proved to be complementary. During the project, the collaboration between taxonomists and the park's scientific services significantly improved. This had positive effects at various levels including improvements of the sampling strategy, better communication with the stakeholders, involvement from local taxonomists and data flow management (Leccia and Morand 2013, Mauz and Granjou 2013).

To complement the taxonomy, molecular analyses were added to the sampling strategy. Barcoding is a standardised method that attributes to each species a unique DNA sequence. The studies were conducted by the Molecular Systematic Service of the MNHN and *Centre de Biologie pour la Gestion des Populations* (Cirad-Ensa-Inra-IRD). The results are published in the sequence database, Barcode of Life Data Systems (Ratnasingham and Hebert 2007) and not presented in this data paper. So far, more than 2,000 gene sequences have been published corresponding to 344 taxa.

### Funding

The ATBI Mercantour/Alpi Marittime was funded via EDIT by the European Commission as part of the sixth framework programme (FP6) between 2006 and 2011. Funds were also provided by France's Ministry of Ecology, the Albert II of Monaco Foundation, the Monegasque Government and the European Regional Development Fund - Alcotra 2007-2013 programme.

Since 2013, the Mercantour National park is continuing the ATBI through different projects and funding sources: Explor'nature bioblitz (Barcelonnette 2017, Sospel 2018, Guillaumes 2019), Wild bees and Myriapods inventories (both funded by the Albert II of Monaco Foundation and the Monegasque Government, 2017-2019 and 2019-2021) and Atlas of biodiversity in the Municipalities (ABC).

Since 2019, a new EU funded ATBI of seven alpine protected areas has been ongoing for 3 years thanks to the Thematic Integrated Plan (PITEM Biodiv'ALP) of the European Territorial Cooperation Programme ALCOTRA (INTERREG).

## Sampling methods

### Sampling description

A great variety of sampling methods were used. Experts could choose their methods, but they had to be accepted by the park authorities prior to the fieldwork. In a limited number of cases, restrictions were applied to specific areas. For example, in the core area of the National Park some methods were prohibited, such as the use of chemicals for surveying earthworms or sampling of rocks covered with saxicolous lichens in archaeological sites. The sampling strategy consisted of a combination of one-shot (individual collecting) and continuous sampling techniques using permanent devices in the field (Deharveng et al. 2015b). The one-shot sampling techniques used either standardised or non-standardised protocols.

Non-standardised individual collecting was the most employed method. It was recognised as one of the most productive methods in terms of species richness because it relies on the expert's field experience (Deharveng et al. 2015b). It is also the easiest protocol to maintain over a long period of time in the context of limited and changing financial and human resources. The major drawback of this approach is the absence of information on the sampling intensity and reproducibility of the methods (Ichter et al. 2014).

**Figure 2.**
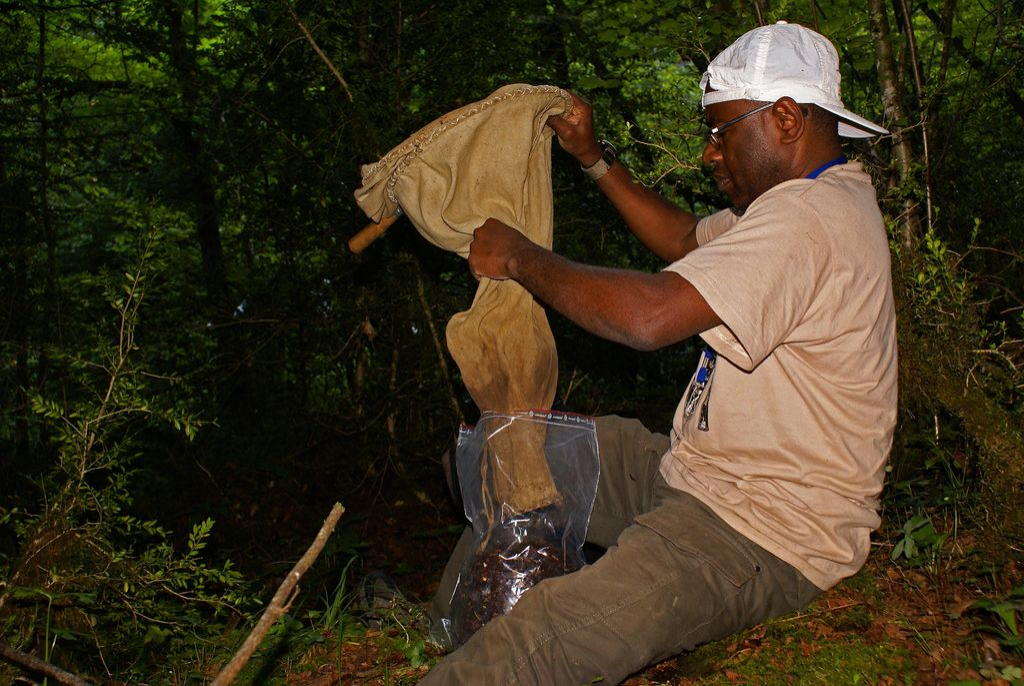
Sieving of soil fauna, A. Abdou (Author: J. Ichter under CC BY-NC-SA).

**Figure 3.**
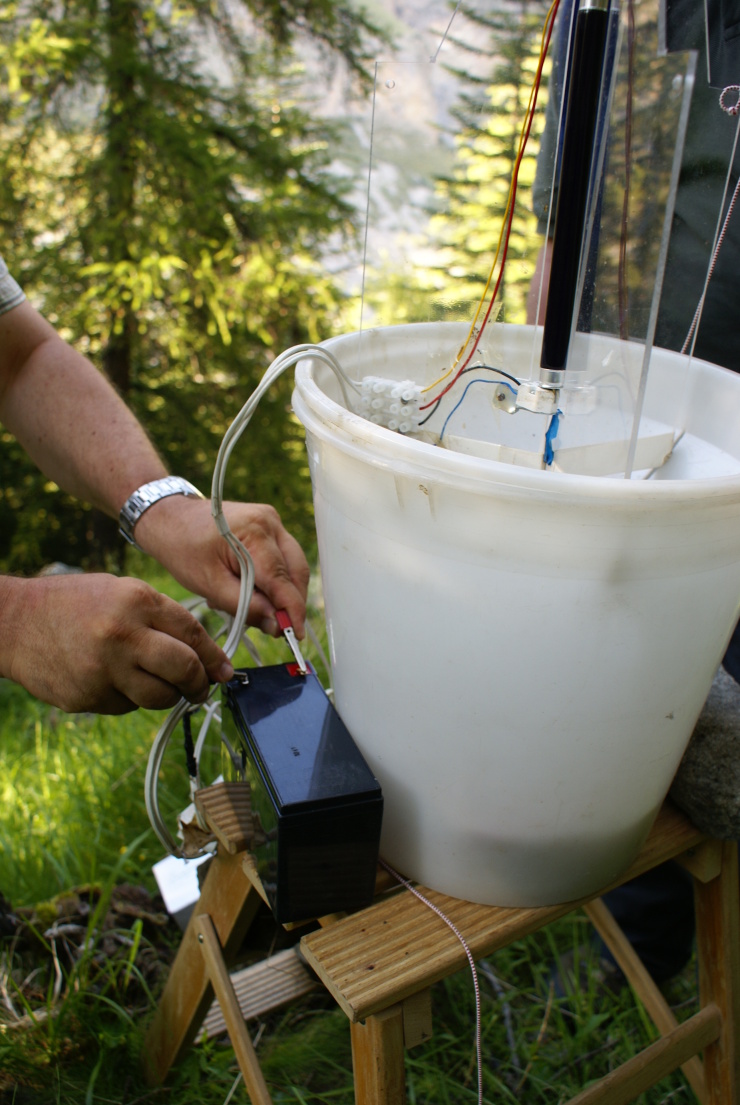
Light traps for butterfly sampling (Author: J. Ichter under CC BY-NC-SA).

The invertebrates provide the most diverse sampling techniques since they target a large spectrum of ecological groups like the flying insects (entomological net, light traps, interception traps), the ground fauna (pitfall traps, soil sieving, see Fig. 2), the terrestrial underground fauna (bait trap), the aquatic insects (artificial susbstrates, nets, light traps, see Fig. 3) and the hyporheic fauna (Bou Rouch filtering or pumping).

The ATBI Mercantour/Alpi Marittime was also an opportunity to collect and disseminate information on methods and protocols. In particular, 79 scholars contributed to the publication of the two volumes of the 'Manual on field recording techniques and protocols for All Taxa Biodiversity Inventories and Monitoring' in ABC taxa, a journal dedicated to capacity building in taxonomy and collection management (Eymann et al. 2010). In 2013, a scientific and technical workshop was organised in the framework of the ATBI and a specific session on methodological issues was held. A summary of the discussions is available in the proceedings (Deharveng and Isaia 2013).

### Quality control

All datasets presented in this publication are managed by the MNHN which is responsible for the national inventory of natural heritage (INPN). The INPN is part of the SINP information system on nature and landscape (http://www.naturefrance.fr) which is the national system for sharing observation data on biodiversity in France. This information system guarantees the traceability of data and authorship and normalised standards of data and metadata.

Before being published, a series of checks are routinely performed (Jomier et al. 2019). The first category of checks is compliance with standard formats of data and metadata. The data must be compliant with both physical and conceptual aspects: mandatory fields, required formats, repositories (including geographical and taxonomical, see Taxonomic coverage), classifications and lists of values. The second category of checks is the consistency to ensure logical compatability within the data, the metadata and between the data and the metadata. For example, the observation start date should always be less than or equal to the observation end date.

In addition, a series of automatic controls called scientific validation were applied to verify that data were compliant with other reference databases: taxonomical repository, biogeographic status and know distribution (e.g. atlas). However, for the Mercantour ATBI, there was no expert validation to assess the reliability, i.e. the degree of confidence that can be placed in the data. The datasets producers are responsible for the reliability of the identification. Authors have the possibility to tag an identification as doubtful, so that the data would not be published.

## Geographic coverage

### Description

The study area covers the territory of the Mercantour National Park (2,163 km²), which is protected and managed as such since 1979.

This territory is divided into two areas: a core area (679 km^2^), which benefits from strict protection and a peripheral area (1,484 km^2^). As biological and geological sampling are forbidden in the core of the National Park, all sampling carried out within the framework of the ATBI has been regulated by specific authorisations. These authorisations were issued to taxonomists upon request after reference check of their skills and reliability.

For this paper, the limits defined for the Mercantour National Park, including core and peripheral areas, are defined by the following communes: Allos (Post Code: 04006), Belvedere (Code: 06013), Beuil (Code: 06016), Breil-Sur-Roya (Code: 06023), Chateauneuf-D'entraunes (Code: 06040), Colmars (Code: 04061), Guillaumes (Code: 06071), Isola (Code: 06073), Jausiers (Code: 04096), La-Bollene-Vesubie (Code: 06020), Larche (Code: 04100), Meyronnes (Code: 04120), Moulinet (Code: 06086), Peone (Code: 06094), Roubion (Code: 06110), Roure (Code: 06111), Saorge (Code: 06132), Sospel (Code: 06136), St-Etienne-De-Tinée (Code: 06120), St-Martin-Vésubie (Code: 06127), St-Sauveur-Sur-Tinée (Code: 06129), Tende (Code: 06163), Uvernet-Fours (Code: 04226), Valdeblore (Code: 06153), Fontan (Code: 06062), Entraunes (Code: 06056), St-Dalmas-Le-Selvage (Code: 06119) and Rimplas (Code: 06102).

**Figure 4.**
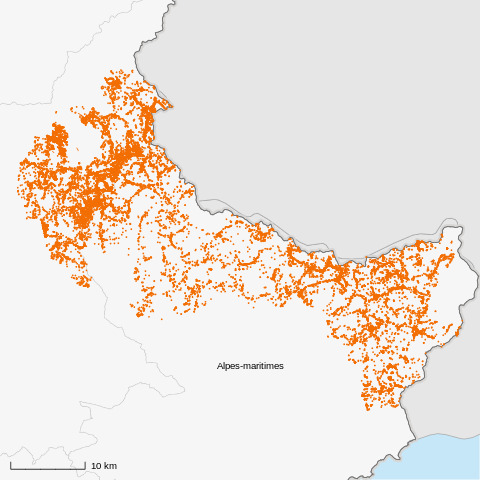
Spatial distribution of the ATBI data (PatriNat, under CC BY-SA).

Fig. 4 illustrates the spatial distribution of the data.

## Taxonomic coverage

### Description

The ATBI Mercantour/Alpi Marittime aims to inventory the entire biota and is mainly focused on four kingdoms: Animalia, Chromista, Fungi and Plantae. For the species occurring in France, the taxonomy follows TAXREF, the national repository for flora, fauna and fungi of metropolitan France and Overseas Territories. TAXREF assigns a unique, unambiguous and (whenever possible) consensual scientific name to all species occurring in France. The repository is constantly updated and a new version is published every year.

The ATBI data package does not include datasets on chordates: Birds, Reptiles, Amphibians, Mammals (managed by the Mercantour National Park information system) and Fishes (managed by the water information system, SIE). However, opportunistic data on chordates were also produced during the inventories and are, therefore, present in the results (except for Fishes).

**Figure 5.**
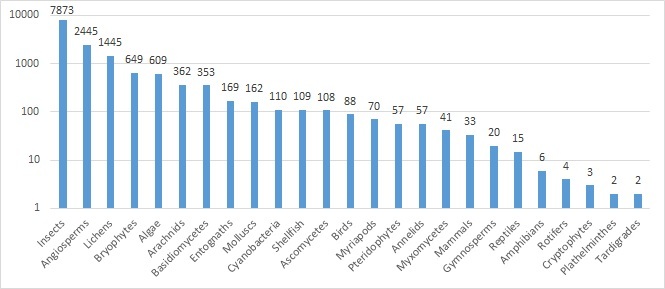
Taxonomic coverage of the inventory: number of taxa per group (Log_10_).

Fig. 5 illustrates the number of species and subspecies per taxonomic/vernacular group on a logarithmic scale.

**Figure 6.**
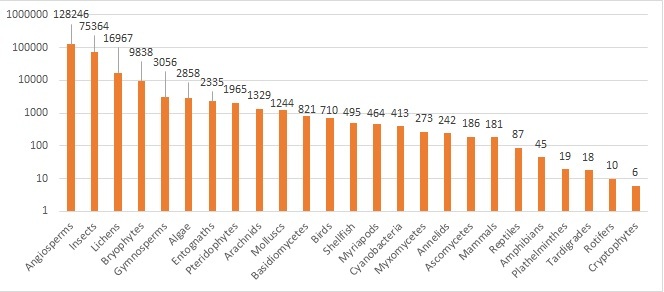
Taxonomic coverage of the inventory: number of occurrences per group (Log_10_) .

Fig. 6 illustrates the number of occurrences per taxonomic/vernacular group on a logarithmic scale.

### Taxa included

**Table:** 

Rank	Scientific Name	
kingdom	Animalia	
kingdom	Chromista	
kingdom	Fungi	
kingdom	Plantae	

## Temporal coverage

### Notes

The ATBI Mercantour/Alpi Marittime officially started in 2007. The data presented here are those collected from this date. It also includes older bibliographic data entered during the project.

In theory, the ATBI will end when the inventory is considered comprehensive. From a technical point of view, an inventory is close to exhaustion when the curve of the number of species inventoried as a function of the sampling effort tends towards a horizontal asymptote, i.e. all species were inventoried at least twice (Fontaine et al. 2012, Ichter et al. 2018). According to the number of new species described and published each year, the authors consider the inventory far from being complete.

Therefore, the ATBI is still on-going due to: 1. successful partnership between the Mercantour National Park and a community of taxonomists and 2. new national and EU-funded projects: Atlas of Biodiversity in the Municipalities and the Thematic Integrated Plan (PITEM Biodiv'ALP) of ALCOTRA Territorial Cooperation Programme.

**Figure 7.**
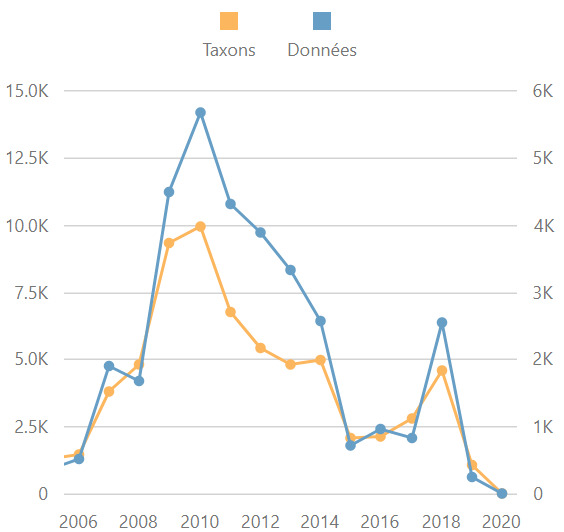
Number of taxa (in yellow) and data (in blue) per year during ATBI programme from 2007 to 2020. Note: this graph only concerns the ATBI main datasets (data from Explor'Nature and Conservatoires botaniques are not included).

**Figure 8.**
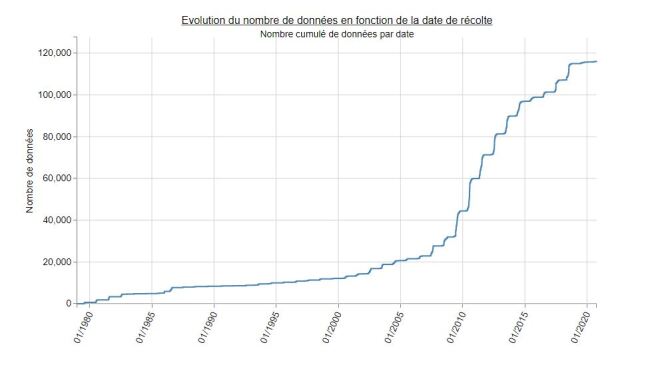
Cumulative number of data according to the sample dates from 1979 to 2020. Note: this graph only concerns the ATBI main datasets (data from Explor'Nature and Conservatoires botaniques are not included).

Fig. 7 shows a temporal distribution of the number of data and taxa per year during the period of the ATBI (2007 - present). Fig. 8 shows the cumulative number of data according to the sample date since the creation of the National Park in 1979. These two graphs only concern data from the main dataset called "Datasets from the Mercantour ATBI". The other datasets from the data package (*cadre d'acquisition*) from Explor'Nature and the Conservatoires botaniques are not included in Figures 7 and 8. The decrease in 2019 and 2020 in Fig. 7 is an artefact due to the delay between the fieldwork and the data publication. Several datasets (e.g. aquatic invertebrates and Syrphids) are expected to be published soon.

## Usage licence

### Usage licence

Creative Commons Public Domain Waiver (CC-Zero)

### IP rights notes

Creative Commons Attribution-Non-Commercial 4.0 International Public Licence

## Data resources

### Data package title

ATBI Parc national du Mercantour

### Resource link

https://inpn.mnhn.fr/espece/cadre/71

### Number of data sets

7

### Data set 1.

#### Data set name

Jeux de données provenant de l'ATBI Mercantour - Datasets from the Mercantour ATBI

#### Data format

Darwin Core Archive

#### Download URL

https://doi.org/10.15468/jtlspu

#### Description

The main dataset of the ATBI in the Mercantour National Park. The project started in 2007 in the framework of the European Distributed Institute of Taxonomy and has continued since 2012 thanks to the collaboration between the Parks, the MNHN and a vast community of taxonomists. It includes data with sampling protocols and opportunistic data collected by taxonomists, park staff and naturalists under a convention with the park and bibliographic data entered as part of the ATBI.

**Data set 1.** 

Column label	Column description
associatedReferences	A list (concatenated and separated) of identifiers (publication, bibliographic reference, global unique identifier, URI) of literature associated with the Occurrence.
basisOfRecord	The specific nature of the data record.
coordinateUncertaintyInMetres	The horizontal distance (in metres) from the given decimalLatitude and decimalLongitude describing the smallest circle containing the whole of the Location. Leave the value empty if the uncertainty is unknown, cannot be estimated or is not applicable (because there are no coordinates). Zero is not a valid value for this term.
country	The name of the country or major administrative unit in which the Location occurs.
countryCode	The standard code for the country in which the Location occurs.
dataGeneralisations	Actions taken to make the shared data less specific or complete than in its original form. Suggests that alternative data of higher quality may be available on request.
datasetID	An identifier for the set of data. May be a global unique identifier or an identifier specific to a collection or institution.
dateIdentified	The date on which the subject was determined as representing the Taxon.
decimalLatitude	The geographic latitude (in decimal degrees, using the spatial reference system given in geodeticDatum) of the geographic centre of a Location. Positive values are north of the Equator, negative values are south of it. Legal values lie between -90 and 90, inclusive.
decimalLongitude	The geographic longitude (in decimal degrees, using the spatial reference system given in geodeticDatum) of the geographic centre of a Location. Positive values are east of the Greenwich Meridian, negative values are west of it. Legal values lie between -180 and 180, inclusive.
eventDate	The date when the Event was recorded (dd/mm/yyyy).
eventID	An identifier for the broader Event that groups this and potentially other Events.
footprintWKT	A Well-Known Text (WKT) representation of the shape (footprint, geometry) that defines the Location. A Location may have both a point-radius representation (see decimalLatitude) and a footprint representation and they may differ from each other.
id	An identifier for the Occurrence (as opposed to a particular digital record of the occurrence). In the absence of a persistent global unique identifier, construct one from a combination of identifiers in the record that will most closely make the occurrenceID globally unique.
identificationVerificationStatus	A categorical indicator of the extent to which the taxonomic identification has been verified to be correct.
identifiedBy	A list (comma separated) of names of people who assigned the Taxon to the subject.
informationWithheld	Additional information that exists, but that has not been shared in the given record.
institutionCode	The name (or acronym) in use by the institution having custody of the object(s) or information referred to in the record.
locality	The specific description of the place.
locationRemarks	Comments or notes about the Location.
maximumDepthInMetres	The greater depth of a range of depth below the local surface, in metres.
maximumElevationInMetres	The upper limit of the range of elevation (altitude, usually above sea level), in metres.
minimumDepthInMetres	The lesser depth of a range of depth below the local surface, in metres.
minimumElevationInMetres	The lower limit of the range of elevation (altitude, usually above sea level), in metres.
modified	The most recent date-time on which the resource was changed.
municipality	The full, unabbreviated name of the next smaller administrative region than county (city, municipality etc.) in which the Location occurs. Do not use this term for a nearby named place that does not contain the actual location.
nameAccordingTo	The reference to the source in which the specific taxon concept circumscription is defined or implied - traditionally signified by the Latin "sensu" or "sec." (from secundum, meaning "according to"). For taxa that result from identifications, a reference to the keys, monographs, experts and other sources should be given.
occurrenceID	An identifier for the Occurrence (as opposed to a particular digital record of the occurrence). In the absence of a persistent global unique identifier, construct one from a combination of identifiers in the record that will most closely make the occurrenceID globally unique.
occurrenceStatus	A statement about the presence or absence of a Taxon at a Location.
originalNameUsage	The taxon name, with authorship and date information if known, as it originally appeared when first established under the rules of the associated nomenclaturalCode. The basionym (botany) or basonym (bacteriology) of the scientificName or the senior/earlier homonym for replaced names.
recordedBy	A list (comma separated) of names of people responsible for recording the original Occurrence. The primary collector or observer is listed first.
scientificName	The full scientific name, with authorship and date information if known.
basisOfRecord	The specific nature of the data record.
taxonID	An identifier for the nomenclatural (not taxonomic) details of a scientific name.

### Data set 2.

#### Data set name

EXPLOR'NATURE 2017, inventaire biologique de la commune de Barcelonnette - EXPLOR'NATURE 2017, biological inventory of the Municipality of Barcelonnette

#### Data format

Darwin Core Archive

#### Download URL

https://doi.org/10.15468/ru5aks

#### Description

Within the framework of its adhesion to the Mercantour National Park, the Municipality of Barcelonnette and the Park organised a 3-day event focused on the knowledge of biodiversity, the discovery of scientific inventories and the exchange with scientists.

**Data set 2.** 

Column label	Column description
idem as "Datasets from the Mercantour ATBI"	idem as "Datasets from the Mercantour ATBI"

### Data set 3.

#### Data set name

EXPLOR'NATURE 2018, inventaire biologique de la commune de Sospel

#### Download URL

https://doi.org/10.15468/s1cjxq

#### Description

Within the framework of its Atlas of Communal Biodiversity, the commune of Sospel and the Mercantour National Park, in partnership and with the financial support of the French Agency for Biodiversity (AFB), organised a 3-day event focused on the knowledge of biodiversity, the discovery of the scientific inventories and the exchange with scientists.

**Data set 3.** 

Column label	Column description
idem as "Datasets from the Mercantour ATBI"	idem as "Datasets from the Mercantour ATBI"

### Data set 4.

#### Data set name

EXPLOR'NATURE 2019, inventaire biologique de la commune de Guillaumes

#### Data format

Darwin Core Archive

#### Download URL

https://doi.org/10.15468/zgdj99

#### Description

Within the framework of its Atlas of Communal Biodiversity, the commune of Guillaumes and the Mercantour National Park, in partnership and with the financial support of the French Agency for Biodiversity (AFB), organised a 3-day event focused on the knowledge of biodiversity, the discovery of the scientific inventories and the exchange with scientists.

**Data set 4.** 

Column label	Column description
idem as "Datasets from the Mercantour ATBI"	idem as "Datasets from the Mercantour ATBI"

### Data set 5.

#### Data set name

Observations floristiques PNM issues de la base de données flore du Conservatoire botanique national alpin - Floristic observations of Mercantour National Park from the flora database of the Conservatoire botanique national alpin.

#### Data format

Darwin Core Archive

#### Download URL

https://doi.org/10.15468/v4dvqb

#### Description

Floristic observations from the flora database of the Conservatoire botanique national alpin (CBNA) carried out by the Parc national du Mercantour (PNM) and located on the territory of the PNM in the Alpes-de-Haute-Provence Department. Observations carried out within the framework of ATBI and other programmes.

**Data set 5.** 

Column label	Column description
idem as "Datasets from the Mercantour ATBI"	idem as "Datasets from the Mercantour ATBI"

### Data set 6.

#### Data set name

Observations floristiques issues de la base de données flore du Conservatoire botanique national alpin - Floristic observations from the flora database of the Conservatoire botanique national alpin.

#### Data format

Darwin Core Archive

#### Download URL

https://doi.org/10.15468/qhwwdf

#### Description

Floristic observations from the flora database of the Conservatoire botanique national alpin (CBNA) in the Mercantour National Park (Alpes-de-Haute-Provence Department). These observations are produced by the CBNA or from the bibliography.

**Data set 6.** 

Column label	Column description
idem as "Datasets from the Mercantour ATBI"	idem as "Datasets from the Mercantour ATBI"

### Data set 7.

#### Data set name

Inventaires du Conservatoire botanique national méditerranéen dans le cadre de l'ATBI Mercantour - Inventories of the Conservatoire botanique national méditerranéen de Porquerolles in the framework of the ATBI Mercantour.

#### Data format

Darwin Core Archive

#### Download URL

https://doi.org/10.15468/qchx42

#### Description

Inventories of the Conservatoire botanique national méditerranéen de Porquerolles carried out between 2008, 2009 and 2010 as part of the ATBI - Mercantour.

**Data set 7.** 

Column label	Column description
idem as "Datasets from the Mercantour ATBI"	idem as "Datasets from the Mercantour ATBI"

## Additional information

### Main results of the ATBI Mercantour

Thanks to the ATBI, 14,791 taxa and 12,640 species are now known from the Mercantour National Park. Between 2007 and 2020, 247,674 data sources were recorded, checked and published in the INPN information system. All this information is available in open access in the GBIF web site.

When compared to the national taxonomic repository (TAXREF v.13), the Mercantour National Park hosts 15% of all species known to occur in metropolitan France in less than 0.4% of the territory (Table 1). This proportion is even higher for several taxonomical groups where the knowledge is considered sufficient, both for the Mercantour and at national level: Bryophytes (42%), Lepidoptera (40%), Orthoptera (40%), Lichens (38%), Odonata (38%), Reptiles (34%) and Plants (25%); see Table 2.

These results confirm the importance of the Mercantour National Park in terms of biodiversity, which several authors consider a hotspot in Europe (Dole-Olivier et al. 2015, Medail and Quezel 1999, Villemant et al. 2015). The ATBI highlights that conservation efforts in the the National Park concern a wide range of taxonomical groups and goes beyond protected and flagship species, such as birds, mammals and vascular plants.

This information can also be used to steer further investigation. Based on the results, the authors consider that a taxonomical group not reaching 15% of the species known to occur in Metropolitan France is likely to be insufficiently inventoried and would require more research. However, the average number of 15% is expected to increase as new results concerning groups such as hemipters, dipters and hymenopters are published in the near future.

### New species to science

Since the beginning of the ATBI, 52 taxa, new to science have been published (see Table 3). It concerns two genera, 47 species and three sub-species. As a comparison, in Europe, 770 new species were described on average each year between 1950 and 2006 (Fontaine et al. 2012). It is a significant contribution for a territory representing 0.025% of the area of Europe.

**Figure 9.**
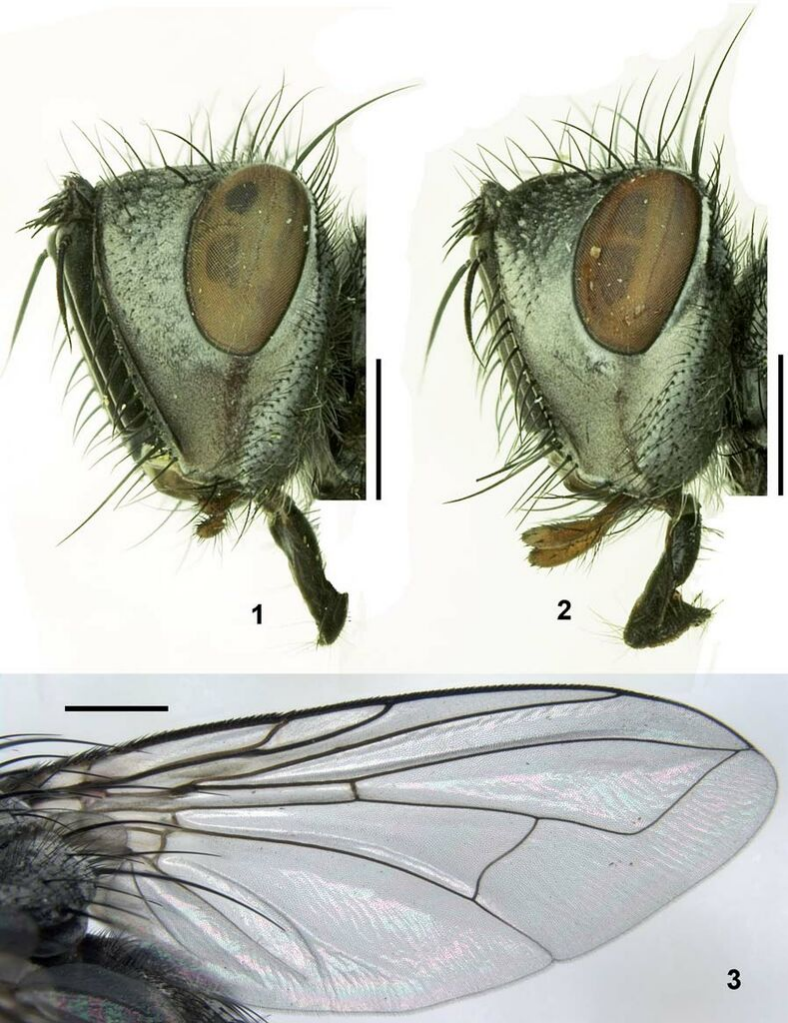
*Istochetaincisor* (Diptera: Tachinidae). Holotype ♂ (Author: H.-P. Tschorsnig, Staatliches Museum für Naturkunde Stuttgart, under copyright).

**Figure 10.**
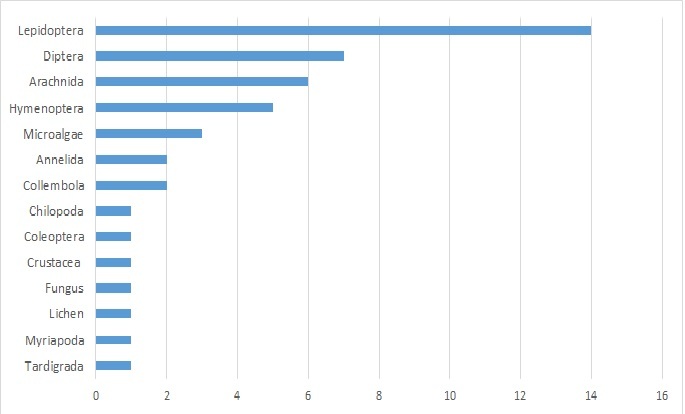
The number of species new to science discovered during the ATBI Mercantour/Alpi Marittime.

About a third of the new species discovered are moth (14 species of Lepidoptera new to science). Flies (Diptera; Fig. 9) represent 15% with seven species new to science. Arachnids (Arachnida) represent 12% with six species new to science. Hymenopters represent 10% with five species. Other discoveries are Microalgae (3 spp.), Annelida (2 spp.), Collembola (2 spp.), Chilopoda (1 sp.), Coleoptera (1 sp.), Crustacea (1 sp.), Fungus (1 sp.), Hemiptera (1 sp.), Lichen (1 sp.) Myriapoda, (1 sp.) and Tardigrada (1 sp.), see Fig. 10.

### Contribution of the ATBI to the knowledge on biodiversity in France

Throughout the duration of the ATBI, 53 species new for France were discovered (see Table 4).

With 247,674 data sources, the ATBI contributes 0.33% of the total amount of data currently released in the INPN (in November 2020). The inventories added 1077 taxa for which no occurrence was previously recorded in the INPN and 1,244 taxa for which no occurrence was previously recorded in the National Park.

### The importance of the information system and long-term preservation of data

The data management strategy is a critical factor for the success of an ATBI project (Langdon et al. 2006). It is a cross-cutting issue that influences the preparation, capture, sharing and use of information (Poncet and Caprio 2013, Ichter et al. 2018). Effective data management at the different stages makes it possible to properly analyse and to ensure they are always accessible and evolving.

The first challenge for the operation of an information system is a common understanding of the sharing rules and procedures by the different partners. This point is all the more important since the various participants often have their own tools and logic according to their resources and objectives. From our experience, data management was largely underestimated (Deharveng et al. 2015b, Villemant et al. 2015) and, as a consequence, part of the data or information on the specimens collected in the field were not entered in the database. For the purpose of this data paper, PatriNat (OFB/MNHN/CNRS) allocated extra resources to enter and publish data with special care for species new to science and species new to France.

The second challenge is interoperability. In the case of the ATBI Mercantour/Alpi Marittime, the cross-border management was an issue since it was not possible to agree on a unique information system for both technical (mainly taxonomical and geographical) and political reasons. Unfortunately, the information systems on both sides of the border are not interoperable. For that reason, this data paper is limited to the French part of the ATBI.

Finally, the long-term preservation of data needs specific infrastructures and resources. By definition, a comprehensive inventory of biodiversity is a long-lasting process. Due to the taxonomic impediment (Fontaine et al. 2012), the results for some of the least-known groups are expected to be published with some delay. Furthermore, taxonomy is a field that is constantly evolving and needs a regular update (see Taxonomic coverage). Therefore, it is necessary to consider the permanence of the information from the beginning of such projects. The first stage of the ATBI Mercantour/Alpi Marittime was coordinated by the EU-funded project EDIT and an ad-hoc database was designed and implemented. At the end of Work package 7, the database and the website have not been maintained, making both rapidly outdated. As a second phase of the project, both parks took the lead of the ATBI and it was decided that, for the French part, the MNHN will be responsible for data management through the INPN. Being recognised as the national information system for biodiversity, the INPN guarantees a long-term preservation of data both technically and scientifically (e.g. evaluation of taxonomy, monitoring of the publications).

### Conclusion

The ATBI Mercantour/Alpi Marittime is the first and one of the most ambitious inventories of its kind in Europe. With 12,640 species registered, the ATBI is the most important inventory in France in terms of species' richness compared to similar initiatives, such as the Réserve naturelle nationale de la Forêt de la Massane in Pyrénées-Orientales (8,200 species in 3.37 km²), the Forêt de Païolive et le plateau des Gras in Ardèche-Gard (5,000 species in 150 km²) and the Réserve intégrale du Lauvitel in the Écrins National Park in Isère (2,200 species in 6.86 km²).

For the Mercantour National Park, the number of species known has doubled since 2007 and it is still growing. The success of the ATBI is the result of four main factors: the extensive sampling over a long period, the key biogeographic location, strong collaboration amongst a wide range of partners and the National Park's administration as the project manager. In terms of management, the discovery of endemic and potentially rare arthropods is a conservation asset similar to large mammals or birds of prey. The ATBI database is regularly used as a tool for a better management of ecosystems, such as forest and grassland. It also provides a consistent framework for future investigation, for example, taxonomic groups, sectors and times to inventory in priority and monitoring schemes. For all those reasons, the Mercantour National Park continues to invest in its ATBI with the support of its partners and a dynamic taxonomist community. The successful experience of the Mercantour/Alpi Marittime is also a benchmark for other national and regional parks (e.g. Ecrins, Vanoise, Queyras) that initiated an ATBI on their territory.

This data package of 247,674 species occurrences with precise information on date, location and altitude is for the first time publicly available for a wide range of uses including scientific investigation, natural area stewardship and conservation policies. More than 1000 scientific publications related to the ATBI Mercantour/Alpi Marittime have already been published (Granjou et al. 2014) and more are expected in the near future. The main outputs concern studies in the field of systematics (taxonomy, phylogenetic, chorology; see Villemant et al. 2015), but also in ecology (e.g. pollination, parasitism, zoochory, food chain; see Lefebvre et al. 2014, La Morgia et al. 2015) and conservation biology (see Bonelli et al. 2015, Villemant et al. 2015).

In the context of the increasing decline in biodiversity, it is more urgent than ever to increase our knowledge on poorly-studied biological groups (McKinney 1999, Sánchez-Bayo and Wyckhuys 2019, Cowie et al. 2022). The risk that species could disappear before being discovered is not only a reality in tropical ecosystems, but also in Europe (Dubois 2003, Mauz 2011, Fontaine et al. 2012). The authors recommend to broaden and replicate the initiative to a series of reference sites in France by taking into account biogeographical and ecosystem representation, including rural and urbanised sites and monitoring schemes. Finally, the information should be used for decision-making and conservation policies, both at local and national level.

## Figures and Tables

**Figure 11.**
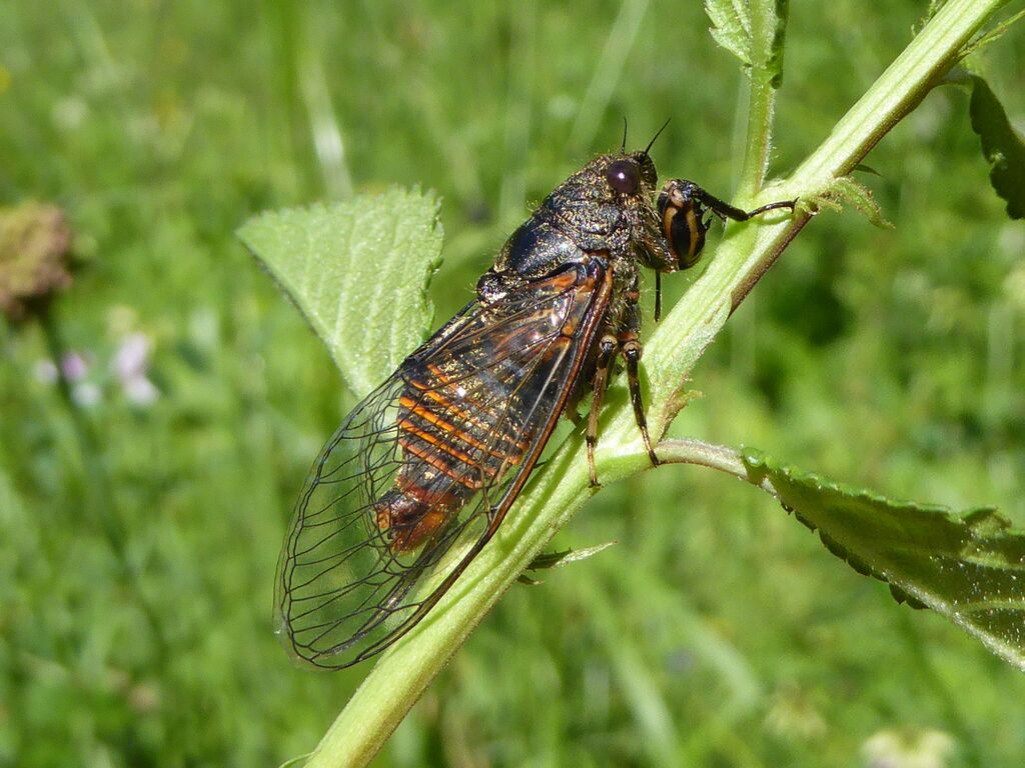
*Cicadettasibillae* (Hemiptera Cicadidae) new cicada species in France (Authors: K. Gurcel, under CC BY-NC-SA).

**Table 1.** Proportion of French species occurring in the Mercantour National Park. Note: numbers above the average proportion are in bold.

			**Species in France**	**Species in Mercantour**	**Proportion**
**Fungi**			24,497	1,651	6.7%
	Lichens		3,165	1,213	**38.3**%
**Algae (sensu lato)**			2,391	414	**17.3**%
**Plants**			10,113	2,530	**25**%
	Bryophytes		1,264	537	**42.5**%
	Angiosperms		7,625	1,697	**22.3**%
	Gymnosperms		73	11	**15.1**%
	Pteridophytes		179	48	**26.8**%
**Animalia**			48,746	8,147	**16.7**%
	Worms		1,376	28	2.0%
	Chordate		857	135	**15.8**%
		Birds	486	86	**17.7**%
		Fishes	81	0	0%
		Reptiles	41	14	**34.1**%
		Amphibians	43	5	11.6%
		Mammals	206	30	14.6%
	Molluscs		700	90	12.9%
	Rotifers		473	1	0.2%
	Arthropods		45,191	7,892	**17.5**%
	including	Insects	39,447	7,333	**18.6**%
		Arachnids	3,481	302	8.7%
		Crustaceans	833	80	9.6%
		Myriapods	524	58	11.1%
		Entognatha	906	119	13.1%
	Tardigrades		67	1	1.5%
**Bacteria**			169	94	**55.6**%
**Protozoa**			525	59	11.2%
**Chromista**			1,396	159	11.4%
**TOTAL**			**85,446**	**12,640**	**14.8**%

**Table 2.** Proportion of the French insects occurring in the Mercantour National Park. Note: numbers above the average proportion are in bold.

**Order**	**Species in France**	**Species in Mercantour**	**Proportion**
Diptera	8,865	761	8.6%
Odonata	98	37	**37.8**%
Mantodea	8	3	**37.5**%
Phasmida	4	1	**25**%
Psocodea	116	0	0%
Blattodea	38	5	13.2%
Hemiptera	3,527	595	16.9%
Mecoptera	10	4	**40**%
Zygentoma	16	0	0%
Coleoptera	10,887	2,086	**19.2**%
Dermaptera	18	5	**27.8**%
Embioptera	2	1	**50**%
Neuroptera	175	39	**22.3**%
Orthoptera	237	95	**40.1**%
Plecoptera	195	56	**28.7**%
Hymenoptera	8,630	1,317	15.3%
Lepidoptera	5,555	2,204	**39.7**%
Megaloptera	3	1	**33.3**%
Trichoptera	473	143	**30.2**%
Siphonaptera	96	7	7.3%
Strepsiptera	13	0	0%
Thysanoptera	265	0	0%
Archaeognatha	52	0	0%
Ephemeroptera	146	31	**21.2**%
Raphidioptera	18	6	**33.3**%
**TOTAL**	**39,447**	**7,397**	**18.8**%

**Table 3.** Species new to science discovered in the framework of the ATBI.

**Species**	**Reference**	**Country where first discovered**	**Collection (Holotype)**
*Dichroramphatarmanni*	Huemer 2009	Italy	coll. P. Huemer
*Clinopodesvesubiensis*	Bonato et al. 2011	France	MNHN, Paris (FR)
*Eulamprotesoccidentella*	Huemer and Karsholt 2011	France	TLMF, Innsbruck (AUS)
*Malthodescoryli*	Liberti 2011	Italy	unknown
*Istochetaincisor*	Tschorsnig 2011	France	SMNS, Stuttgart (GER)
*Klimeschiopsismaritimaealpina*	Nel and Varenne 2011	France	TLMF, Innsbruck (AUS)
*Sistotremaampullaceum*	Duhem 2011	Italy	unknown
*Troglocheleslanai*	Zacharda et al. 2011)	Italy	MBD-OSU, Columbus (USA)
*Duvaliusmagdelaineitordjmani*	Lemaire and Raffaldi 2011	France	MNHN, Paris (FR)
*Caryocolumdauphini*	Grange and Nel 2012	France	MNHN, Paris (FR)
*Diplocephalusguidoi*	Frick and Isaia 2012)	Italy	MCSNB, Bergamo (IT)
*Histoponaleonardoi*	Bolzern et al. 2013	Italy	MCSNB, Bergamo (IT)
*Eulamprotesmirusella*	Huemer et al. 2013)	France	TLMF, Innsbruck (AUS)
*Plinthisusheteroclitus*	Matocq and Pluot-Sigwalt 2013	France	MNHN, Paris (FR)
*Bryobiacinereae*	Auger et al. 2015	France	CBGP, Montpellier (FR)
*Bryobiamercantourensis*	Auger et al. 2015	France	CBGP, Montpellier (FR)
*Eotetranychusquercicola*	Auger et al. 2015	France	CBGP, Montpellier (FR)
*Aberrantidrilus*	Martin et al. 2015	France	MNHN, Paris (FR)
*Aberrantidrilusstephaniae*	Martin et al. 2015	France	MNHN, Paris (FR)
*Alloxystaalpina*	Ferrer-Suay et al. 2015	France	MNHN, Paris (FR)
*Alloxystafranca*	Ferrer-Suay et al. 2015	France	MNHN, Paris (FR)
*Alloxystapilae*	Ferrer-Suay et al. 2015	France	MNHN, Paris (FR)
*Deutonurajeromoltoi*	Deharveng et al. 2015a	France	MNHN, Paris (FR)
*Echiniscuspardalis*	Degma and Schill 2015	Italy	coll. R. Schill
*Empisfusca*	Daugeron and Lefebvre 2015	France	MNHN, Paris (FR)
*Marioninasambugarae*	Martin et al. 2015	France	MNHN, Paris (FR)
*Nematopogonargentellus*	Leraut and Leraut 2015	France	MNHN, Paris (FR)
*Odontidiumapiculatum*	Jüttner et al. 2015	Italy	NMW, Cardiff (UK)
*Odontidiumneolongissimum*	Jüttner et al. 2015	Italy	Herb. Mus. Palat. Vindob., Acqu.
	Jüttner et al. 2015	Italy	coll. F. Meister, Engadin (CH)
*Orogastruratetrophthalma*	Deharveng et al. 2015a	France	MNHN, Paris (FR)
*Rhamphomyiabrevis*	Daugeron and Lefebvre 2015	France	MNHN, Paris (FR)
*Xyalaspispseudolaevigata*	Mata-Casanova et al. 2015	France	MNHN, Paris (FR)
*Cricotopusroyanus*	Moubayed-Breil 2016	France	coll. J. Moubayed-Breil, Montpellier (FR)
*Dichroramphamelaniana*	Nel and Varenne 2016	France	Coll. Th. Varenne, Nice (FR)
*Kesslerialativalva*	Nel and Varenne 2016	France	Coll. Th. Varenne, Nice (FR)
*Mercantouria*	Huemer et al. 2016	France	TLMF, Innsbruck (AUS)
*Mercantourianeli*	Huemer et al. 2016	France	TLMF, Innsbruck (AUS)
*Stomopteyrxalpinella*	Nel and Varenne 2016	France	Coll. Th. Varenne, Nice (FR)
*Autaretiaaliciae*	Geoffroy and Mauries 2017	France	MNHN, Paris (FR)
*Chaetocladiuscoppai*	Moubayed-Breil and Dia 2017	France	coll. J. Moubayed-Breil Montpellier (FR)
*Polypedilummercantourus*	Moubayed-Breil 2017	France	coll. J. Moubayed-Breil Montpellier (FR)
*Virgatanytarsusrossaroi*	Moubayed-Breil 2017	France	coll. J. Moubayed-Breil Montpellier (FR)
*Agrotismayrorum*	Ronkay and Huemer 2018	France	TLMF, Innsbruck (AUS)
*Grammospilamartae*	van Achterberg 2018	Italy	RMNH, Leiden (NL)
*Setinairrorellapanthera*	Leraut 2018	France	MNHN, Paris (FR)
*Acarosporaepiaspicilia*	Roux et al. 2019	France	Coll. C. Roux, Mirabeau (FR)
*Stygepactophanesoccitanus*	Galassi et al. 2019	France	MNHN, Paris (FR)
*Caryocolumlamai*	Huemer 2020	France	TLMF, Innsbruck (AUS)
*Caryocolumhabeleri*	Huemer 2020	France	TLMF, Innsbruck (AUS)
*Kessleriahelveticalecciae*	Leraut 2020	France	MNHN, Paris (FR)
*Scrobipalpahuemeri*	Leraut 2020)	France	MNHN, Paris (FR)

Acronyms. CBGP: Centre de Biologie pour la Gestion des Populations (UMR INRA, CIRAD, IRD, Montpellier SupAgro); MBD-OSU: Museum of Biological Diversity-Ohio State University; MCSNB: Museo Civico di Storia Naturale Bergamo; MNHN: Muséum national d'Histoire naturelle; NMW: National Museum of Wales; RMNH: Naturalis Biodiversity Center; SMNS: Staatliche Museum für Naturkunde Stuttgart; TLMF: Tiroler Landesmuseum Ferdinandeum.

**Table 4.** Species new for France discovered in the framework of the ATBI.

Species	Taxon authorities	Reference
*Catocalalupina*	Herrich-Schäffer, 1851	Tautel and Barbut 2009
*Moehringiaargenteria*	Casazza & Minuto, 2008	comm. pers. Noble (2009)
*Mimelaaurata*	(Fabricius, 1801)	Vincent and Ponel 2009
*Drosophilasuzukii*	(Matsumura, 1931)	Calabria et al. 2010
*Ceratophyllusvagabundusalpestris*	Jordan, 1926	Lemaire and Beaucournu 2012
*Geocorisphaeopterus*	(Germar, 1838)	Maurel and Streito 2012
*Dicyphusflavoviridis*	Tamanini, 1949	Matocq and Streito 2013
Helicoconis (Fontenellea) hispanica	Ohm, 1965	Tillier 2013
Helicoconis (Helicoconis) hirtinervis	Tjeder, 1960	Tillier 2013
*Hydrocyphonovatus*	Nyholm, 1967	Queney 2014
*Nomadagransassoi*	SCHWARZ, 1986	Dufrêne et al. 2014
*Platycheirusciliatus*	Bigot, 1884	Ssymank and Lair 2014
*Platycheirusfasciculatus*	Loew, 1856	Ssymank and Lair 2014
*Alloxystaabdera*	Fergusson, 1986	Ferrer-Suay et al. 2015
*Alloxystaarcuata*	(Kieffer, 1902)	Ferrer-Suay et al. 2015
*Alloxystabrachycera*	Hellén, 1963	Ferrer-Suay et al. 2015
*Alloxystabrevis*	(Thomson, 1962)	Ferrer-Suay et al. 2015
*Alloxystafracticornis*	(Thomson, 1862)	Ferrer-Suay et al. 2015
*Alloxystamullensis*	(Cameron, 1883)	Ferrer-Suay et al. 2015
*Alloxystapilipennis*	(Hartig, 1840)	Ferrer-Suay et al. 2015
*Alloxystapostica*	(Hartig, 1841)	Ferrer-Suay et al. 2015
*Alloxystaproxima*	Belizin, 1962	Ferrer-Suay et al. 2015
*Apocharipstrapezoidea*	(Hartig, 1841)	Ferrer-Suay et al. 2015
*Bactericeraparastriola*	Conci, Ossiannilsson & Tamanini, 1988	Ouvrard et al. 2015
*Cacopsyllapropinqua*	(Schaefer, 1949)	Ouvrard et al. 2015
*Craspedoleptaartemisiae*	(Foerster, 1848)	Ouvrard et al. 2015
*Craspedoleptanebulosa*	(Zetterstedt, 1828)	Ouvrard et al. 2015
*Cyamophilaprohaskai*	(Priesner, 1927)	Ouvrard et al. 2015
*Triozaflixiana*	Burckhardt & Lauterer, 2002	Ouvrard et al. 2015
*Triozasenecionis*	(Scopoli, 1763)	Ouvrard et al. 2015
*Triozaflixiana*	Burckhardt & Lauterer, 2002	Ouvrard et al. 2015
Lasioglossum (Dialictus) duckei	(Alfken, 1909)	Pauly et al. 2015
*Phaenoglyphisabbreviata*	(Thomson, 1877)	Ferrer-Suay et al. 2015
*Phaenoglyphisamericana*	Baker, 1896	Ferrer-Suay et al. 2015
*Phaenoglyphiscalverti*	Andrews, 1978	Ferrer-Suay et al. 2015
*Phaenoglyphisevenhuisi*	Pujade-Villar & Paretas-Martínez, 2006	Ferrer-Suay et al. 2015
*Phaenoglyphisfuscicornis*	(Thomson, 1877)	Ferrer-Suay et al. 2015
*Phaenoglyphisgutierrezi*	Andrews, 1978	Ferrer-Suay et al. 2015
*Phaenoglyphislongicornis*	(Hartig, 1840)	Ferrer-Suay et al. 2015
*Nematopogonsericinellus*	Zeller, 1847	Leraut and Leraut 2015
*Drepanepteryxalgida*	(Erichson in Middendorff, 1851)	Tillier 2015
*Chrysotoxumtomentosum*	Giglio-Tos, 1890	Speight et al. 2016
*Stelisfranconica*	BLÜTHGEN 1930	Genoud and Dufrêne 2016
*Apataniazonella*	(Zetterstedt, 1840)	Coppa and Le Guellec 2017
*Cheilosiarhodiolae*	Schmid, 2000	Speight et al. 2017
*Cionusleonhardi*	Wingelmüller, 1914	Bouyon 2018
*Acompsiasubpunctella*	Svensson, 1966	Leraut and Leraut 2018
*Cicadettasibillae* Fig. 11	Hertach & Trilar, 2015	Puissant and Gurcel 2018
*Epiblemaconfusana*	(Herrich-Schäffer, 1856)	Leraut and Leraut 2018
*Scrobipalpaclintoni*	Povolný, 1968	Leraut and Leraut 2018
*Chryssonordica*	(Chamberlin & Ivie, 1947)	Milano et al. 2019
*Urozelotestrifidus*	Tuneva, 2003	Milano et al. 2019
*Hoplodrinaalsinides*	Costantini, 1922	Huemer et al. 2020
